# Safety and pharmacokinetics of DS-6051b in Japanese patients with non-small cell lung cancer harboring *ROS1* fusions: a phase I study

**DOI:** 10.18632/oncotarget.25263

**Published:** 2018-05-04

**Authors:** Yutaka Fujiwara, Masayuki Takeda, Noboru Yamamoto, Kazuhiko Nakagawa, Kaname Nosaki, Ryo Toyozawa, Chihiro Abe, Ryota Shiga, Kenji Nakamaru, Takashi Seto

**Affiliations:** ^1^ Department of Experimental Therapeutics, National Cancer Center Hospital, Chuo-ku, Tokyo 104-0045, Japan; ^2^ Department of Medical Oncology, Kindai University Faculty of Medicine, Osaka-Sayama-shi, Osaka 589-8511, Japan; ^3^ Department of Thoracic Oncology, National Hospital Organization Kyushu Cancer Center, Minami-ku, Fukuoka-shi, Fukuoka 811-1395, Japan; ^4^ Daiichi Sankyo Co., Ltd., Hiromachi, Shinagawa-ku, Tokyo 140-8710, Japan

**Keywords:** DS-6051b, non-small cell lung cancer, ROS1, pharmacokinetics, Japanese

## Abstract

Oncogenic *ROS1* and *NTRK* fusions were reported in solid tumors, including non-small cell lung cancer (NSCLC). DS-6051b is an oral, potent selective small molecule tyrosine kinase inhibitor. We report the safety, tolerability, efficacy, and pharmacokinetics of DS-6051b in 15 Japanese patients with NSCLC harboring *ROS1* fusions. Patients received DS-6051b once daily (400 mg *n* = 6; 600 mg *n* = 6; or 800 mg *n* = 3) for cycles of 3 weeks. Safety, tolerability, maximum-tolerated dose, pharmacokinetics, and recommended dose for phase II were determined. Common treatment-related adverse events were increased: aspartate aminotransferase and alanine aminotransferase (80.0% each), diarrhea (53.3%), and nausea (46.7%). Dose-limiting toxicities (two grade-3 alanine aminotransferase increases) were seen in the 800 mg cohort. The maximum-tolerated dose and recommended phase II dose was 600 mg once daily. Plasma concentrations of free DS-6051b and DS-6051a increased with dose. Compared with a US phase I study, AUC_0–24 h_ on day 15 was higher but narrowed after body weight correction. Objective response rate was 58.3% in patients with target lesions (*n* = 12) and 66.7% in crizotinib-naïve patients (*n* = 9). Disease control rate was 100%. DS-6051b is well tolerated and effective in Japanese patients with NSCLC harboring *ROS1* fusions and might be a targeted therapy for advanced NSCLC.

## INTRODUCTION

Driver gene mutations and chromosomal gene rearrangements lead to oncogenic tyrosine kinase activation [1, 2]. Fusions of the ROS proto-oncogene 1, receptor tyrosine kinase gene (*ROS1*) and the neurotrophic receptor tyrosine kinase (*NTRK*) genes *NTRK1*, *NTRK2*, and *NTRK3* are some of the key fusion kinases driving cellular transformation in several malignancies. *ROS1* fusion has been reported in glioblastoma multiforme [3, 4], non-small cell lung cancer (NSCLC) [5], cholangiocarcinoma [6], gastric cancer [7], and colorectal cancer [8], and NTRK fusion proteins have been reported in thyroid carcinoma [9], colorectal cancer [10], melanoma [11], breast cancer [12], and NSCLC [13, 14].

*ROS1* rearrangements are found in approximately 1%–2% of NSCLC patients [15, 16]. Previously, the ROS1 inhibitor crizotinib showed a high response rate (objective response rate [ORR] of 72%; median duration of response: 17.6 months, *n* = 50) [17], and it is approved globally for use in advanced NSCLC patients with *ROS1* fusions. However, resistance to crizotinib, caused by a G2032R or other mutation within the kinase domain, has also been reported in *ROS1* fusion-positive NSCLC [18]. A phase II study reported that ceritinib, an anaplastic lymphoma kinase and ROS1 inhibitor, had efficacy in patients with ROS1-rearranged NSCLC who were previously treated with multiple chemotherapy [19].

Drugs targeting fusion kinases are an emerging paradigm in the management of these cancers. DS-6051b is an orally available small molecule receptor tyrosine kinase inhibitor with high affinity for ROS1, NTRK1, NTRK2, and NTRK3 receptors and suppression of their activity *in vitro* [20]. DS-6051b also has antitumor activity in glioblastoma cell lines harboring a *ROS1* fusion, and in human colorectal cancer cell lines harboring a *NTRK1* fusion [20]. An *in vivo* antitumor effect has been demonstrated in a mouse model grafted with the respective tumor cell lines [20]. DS-6051b inhibits the intracellular phosphorylation of ROS1 and NTRK1 in a concentration-dependent manner. It also inhibits the growth of *ROS1*- and *NTRK1*-fusion tumor xenografts in a dose-dependent manner without causing severe body weight loss. A major advantage of DS-6051b over other compounds is that it is also effective against crizotinib-resistant *ROS1* mutations and inhibits the growth of Ba/F3 cells expressing *NTRK* gene rearrangements, both *in vitro* and *in vivo* [20]. In addition to DS-6051b, other drugs in development for cancer treatment include larotrectinib (LOXO101), an NTRK inhibitor, and entrectinib (RXDX101), an NTRK/ROS1 inhibitor.

Two phase I studies for DS-6051b are ongoing in the US and Japan. An open-label, multiple-dose, first-in-human study of DS-6051b in subjects with advanced solid tumors is in progress in the US (U101, NCT02279433) [21]. Here, we report the results of a phase I study evaluating the safety, tolerability, efficacy, and pharmacokinetics (PK) of DS-6051b administered as monotherapy with once-daily multiple oral doses in Japanese patients with solid tumors harboring *ROS1* or *NTRK* fusions (J102, NCT02675491).

## RESULTS

### Patients

A total of 15 patients were enrolled between February 2016 and February 2017. All patients had solid tumors and were diagnosed with *ROS1* fusion-positive NSCLC. Patients started DS-6051b at doses of 400 mg (*n = 6*) or 800 mg (*n* = 3) once daily (QD). An additional cohort of 600 mg QD (*n = 6*) was enrolled to determine the maximum-tolerated dose (MTD) (Supplementary Figure 1). The patient demographics are shown in Table 1. No patient tested positive for *NTRK* fusion. Prior systemic therapy was reported in 15 patients (median [range]: 2 [1–11] regimens). Regarding pretreatment with crizotinib, 3 of 4 patients showed disease progression, and one discontinued treatment because of an adverse event (AE). In addition, one of the crizotinib pretreatment patients who had a PR of DS-6051b discontinued crizotinib treatment because of an AE; therefore, they ceased treatment because of a lack of tolerability to crizotinib.

**Table 1:** Patient demographics and characteristics

Characteristics	Overall (*N* = 15)
Age, median (range)	51 (34–69)
Sex, *n* (%)	
Male	8 (53.3)
Female	7 (46.7)
ECOG PS, *n* (%)	
PS 0	9 (60.0)
PS 1	6 (40.0)
Tumor type, *n* (%)	
Non-small cell lung cancer: adenocarcinoma	15 (100)
With brain metastases, *n* (%)	5 (33.3)
Gene rearrangements, *n* (%)	
*ROS1*	15 (100)
*NTRK1–3*	0
*ROS1* detection method^*^	
FISH	10 (66.7)
RT-PCR	13 (86.7)
Next generation sequencing	3 (20.0)
Previous regimens, *n* (%)	
1	7 (46.7)
≥2	8 (53.3)
Prior crizotinib treatment	4 (26.7)

Abbreviations: ECOG PS, Eastern Cooperative Oncology Group Performance Status; FISH, Fluorescence *in situ* hybridization; RT-PCR, Reverse transcription polymerase chain reaction.

^*^ROS1 was detected by multiple methods for each patient.

### Safety

Investigator-determined treatment-related AEs occurring in at least 20% of patients are listed in Table 2. The most common treatment-related AEs were aspartate aminotransferase (AST) increased (80.0%), alanine aminotransferase (ALT) increased (80.0%), diarrhea (53.3%), nausea (46.7%), creatinine increased (33.3%), and constipation (33.3%). Two dose-limiting toxicities (DLTs) (grade 3 ALT increase) were seen in patients receiving 800 mg DS-6051b. No DLTs occurred in the 400 mg and 600 mg cohorts. The MTD and the recommended phase II dose was determined to be 600 mg QD.

**Table 2:** Safety and tolerability: treatment-related adverse events observed in ≥20% subjects and hepatotoxicity adverse events

Adverse Event, *n* (%)		400 mg (*n* = 6)		600 mg (*n* = 6)			800 mg (*n* = 3)		Overall (*n* = 15)
ALT increased		4 (66.7)		5 (83.3)			3 (100.0)		12 (80.0)
AST increased		4 (66.7)		5 (83.3)			3 (100.0)		12 (80.0)
Diarrhea		3 (50.0)		3 (50.0)			2 (66.7)		8 (53.3)
Nausea		1 (16.7)		4 (66.7)			2 (66.7)		7 (46.7)
Blood creatinine increased		2 (33.3)		2 (33.3)			1 (33.3)		5 (33.3)
Constipation		3 (50.0)		1 (16.7)			1 (33.3)		5 (33.3)
Decreased appetite		1 (16.7)		1 (16.7)			1 (33.3)		3 (20.0)
Dysgeusia		1 (16.7)		1 (16.7)			1 (33.3)		3 (20.0)
Malaise		0		2 (33.3)			1 (33.3)		3 (20.0)
Vomiting		2 (33.3)		0			1 (33.3)		3 (20.0)
**Adverse Event, *n* (%)**	**Grade 1/2**		**Grade 3**		**Grade 4**	**All subjects**	
ALT increased	9 (75.0)		3 (25.0)		0	12	
AST increased	11 (91.7)		1 (8.3)		0	12	

Abbreviations: ALT, alanine aminotransferase, AST, aspartate aminotransferase.

The two most frequent adverse events are also represented by grade.

Three patients developed serious AEs, including drug-related grade 3 retinal detachment (*n* = 1), drug-related grade 5 interstitial lung disease (*n* = 1), and grade 2 pneumothorax (*n* = 1). There were eleven ≥ grade 3 AEs (three ALT increased, one AST increased, one retinal detachment, one case of interstitial lung disease, one creatine phosphokinase increased, one hypoalbuminemia, one case of anemia, one white blood cell count decreased, and one neutrophil count decreased) experienced by eight of the 15 patients (53.3%).

Two patients withdrew from the study due to toxicity, one because of a grade 3 AST/ALT increase, and one because of grade 5 interstitial lung disease. The latter patient had a pretreatment history of carboplatin + bevacizumab + pemetrexed and avelumab, and this event occurred 40 days after the first 400 mg dose of DS-6051b. Despite treatment with prednisolone, the patient died after 2 months. The death was deemed due to progressive disease, but it may be attributable to interstitial lung disease.

Dose interruptions were reported in seven patients who received 400 mg, 600 mg, or 800 mg DS-6051b and were due to grade 3 ALT increased (*n* = 2), grade 2 ALT increased (*n* = 1), grade 2 AST increased (*n* = 1), grade 2 pneumothorax (*n* = 1), grade 3 retinal detachment (*n* = 1), grade 4 creatine phosphokinase increased (*n* = 1), or grade 4 neutrophil count decreased (*n* = 1).

### PK endpoints

**Figure 1:**
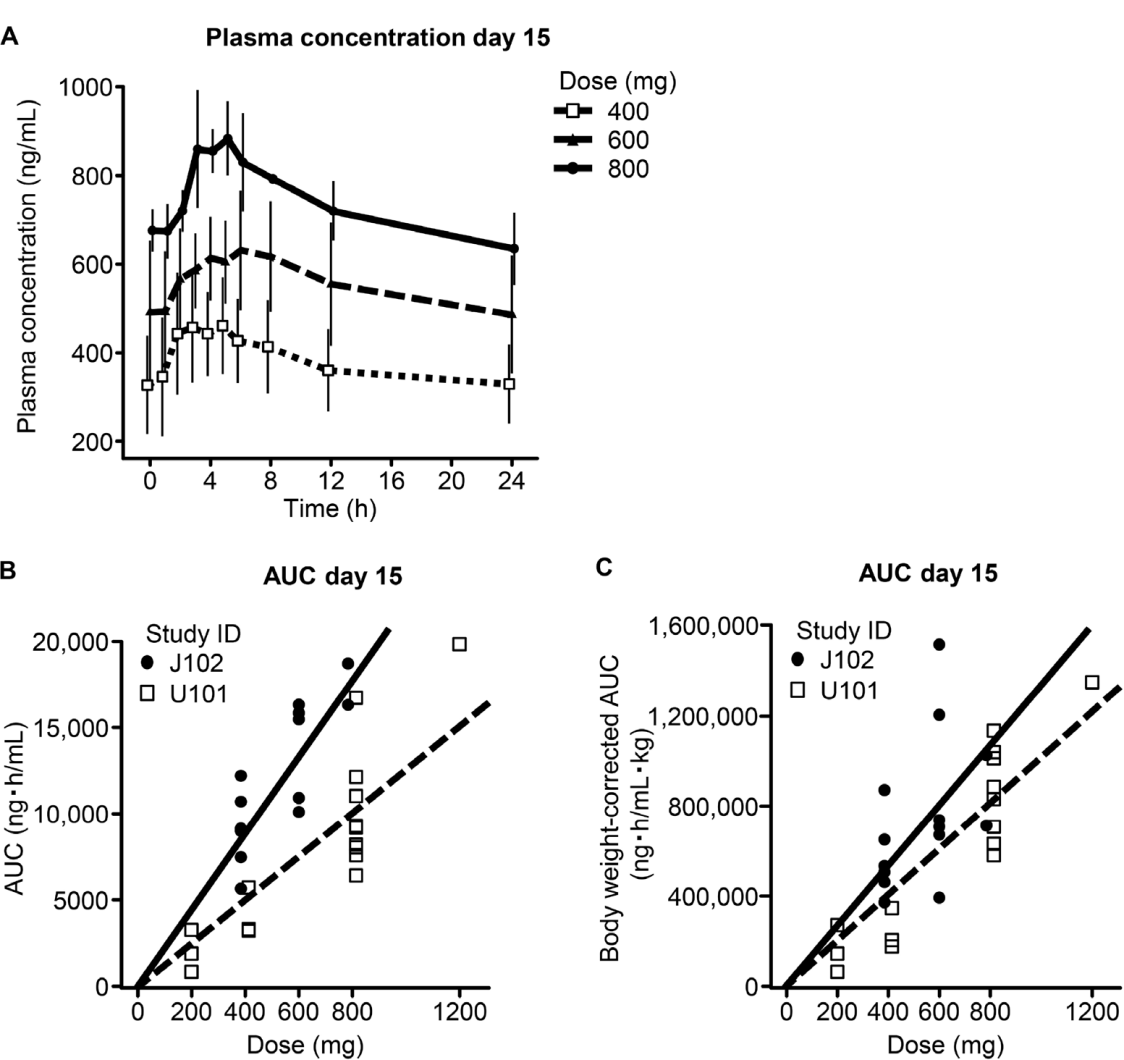
Pharmacokinetic profile Plasma concentration curves for DS-6051b (**A**). Comparison of AUC_0–24 h_ in the current study and the US phase I study (U101) without body weight correction (**B**) and with body weight correction (**C**). The regression coefficient (slope) is 1.77 times as steep as in the US study but this difference decreases to 1.32 times when corrected for body weight.

The plasma concentration of DS-6051a increased in a dose-dependent manner (Figure 1A). The geometric mean C_max_ at day 15 for 400 mg and 800 mg DS-6051b were 469 ng/mL and 886 ng/mL, respectively. The median T_max_ was 4.0 h for both 400 mg and 800 mg.

The geometric mean AUC_0–24 h_ on day 15 was 8770 ng∙h/mL for 400 mg and 17500 ng∙h/mL for 800 mg. The regression coefficient (slope) was 1.77 times as steep as that observed in the US study (Figure 1B), but decreased to 1.32 times after correction for body weight (Figure 1C). The overall pharmacokinetic profile of DS-6051b is shown in Supplementary Table 1.

### Efficacy endpoints

**Figure 2:**
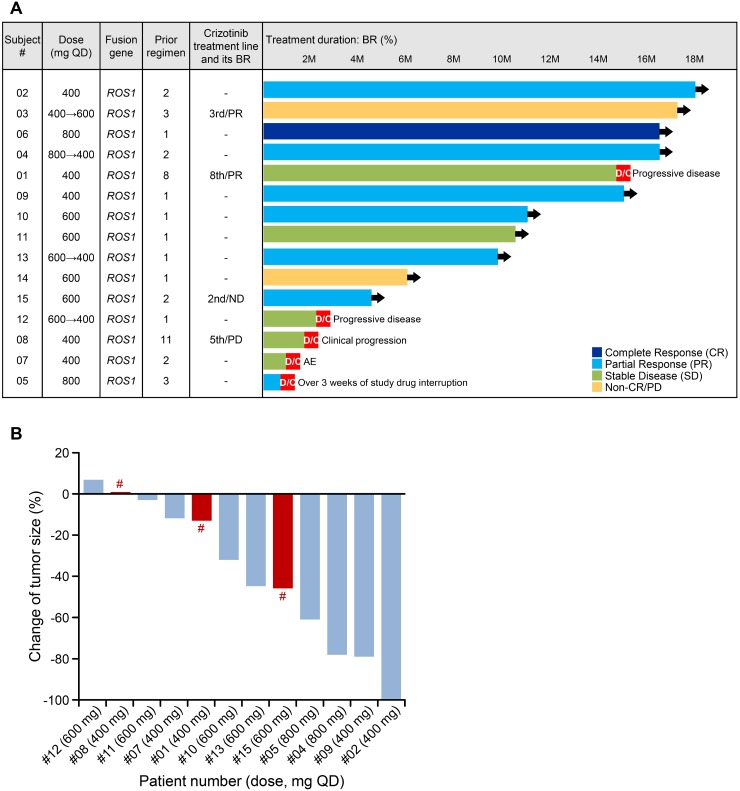
Efficacy endpoints Efficacy of DS-6051b (*n* = 15; cut-off date: 6 July 2017) (**A**). Best percentage change of tumor size from baseline in patients with target lesion (**B**). ^#^Crizotinib pre-treated patient. Non-CR/non-PD: persistence of one or more non-target lesion(s) and/or maintenance of tumor marker level above the normal limits; BR, best response; QD, once daily; M, month (4 weeks); D/C: discontinued. Black arrow (**➔**) indicates ongoing treatment.

Of the 15 patients, 12 had measurable tumor lesions and of those, nine were crizotinib treatment naïve. The ORRs were 58.3% (seven partial responses [PRs]) and 66.7% (six PRs), respectively. The disease control rate (DCR) was 100% in both groups (Figure 2A). Tumor size reduction was observed in all dose groups (Figure 2B). The median treatment duration was 10 months with one patient able to continue for more than 18 months (#02). In the 800 mg cohort, one of the three patients with no target lesion had a complete response (CR) (Figure 2A). Among crizotinib-pretreated patients, the ORR was 33.3% (one PR) and the DCR was 100% (one PR, two with stable disease (SD)). Three patients underwent a dose reduction because of one of the following: patient request and malaise, increased ALT level, or malaise (Figure 2A).

**Figure 3:**
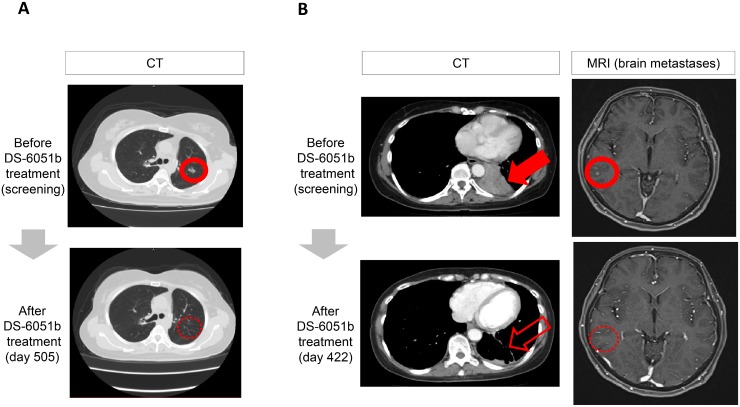
Computed tomography (CT) and magnetic resonance images (MRI) for objective responses (**A**) Patient #02: 400 mg QD, 43-year-old female, ROS1 + non-small cell lung cancer (NSCLC). Best response: partial response, crizotinib treatment-naïve. This patient showed a partial response from day 43 (showing a best response of −100% from day 127). (**B**) Patient #09: 400 mg QD, 61-year-old female, ROS1 + NSCLC. Best response: partial response, crizotinib treatment-naïve. This patient showed a partial response from day 44 (showing a best response of −79% from day 338) and her brain metastatic lesion disappeared. QD, once daily.

In the 400 mg cohort, a 43-year-old female patient (#02) showed a PR (best response of a 100% reduction in tumor size from baseline) from day 43 and best response from day 127 (Figure 3A). Another 61-year-old crizotinib-naïve female patient (#09) attained a PR (best response of a 79% reduction in tumor size from baseline) and her brain metastatic lesions disappeared (Figure 3B).

## DISCUSSION

This study is the first report examining the safety and tolerability, as well as dose setting and efficacy, of DS-6051b for *ROS1* fusion-positive NSCLC. With two phase I studies for DS-6051b in the US and Japan (NCT02279433; NCT02675491), this study is part of a comprehensive worldwide clinical development program.

In this phase I study, the MTD and the recommended phase II dose of DS-6051b was determined to be 600 mg QD. In the US phase I trial, the MTD was confirmed to be 800 mg QD [21]. The main reason for this discrepancy between the US and Japan is the difference in exposure to DS-6051b, as a higher AUC_0–24 h_ on day 15 was observed in this study, although this decreased when corrected for body weight.

The AE profile of DS-6051b included abnormalities of liver and gastrointestinal functions. ALT and AST increased were observed in 80.0% of patients. Although the two DLTs observed were both grade 3 ALT increased in patients receiving 800 mg, and two patients withdrew from treatment due to grade 3 AST and ALT increased, most of the hepatic AEs were grade 2 or less, and no grade 4 AE was observed. Diarrhea and nausea occurred in about half of patients, but both were grade 2 or less and did not require dose interruption. In the crizotinib study, which also targeted *ROS1* fusions, high rates (82%) of visual disturbance were reported [17], but we observed few cases of visual disturbance in our study.

The incidence of increased ALT was higher in this study (12/15 patients [80%]) compared with the US phase I trial (1/19 patients [5.3%]) at the same dose (doses up to 800 mg). The incidence of increased AST was similarly higher (also 80% vs 5.3%) [21]. Diarrhea and nausea were common treatment-related AEs in both studies. Overall, DS-6051b was well tolerated in Japanese patients with NSCLC.

Regarding the efficacy of DS-6051b, ORR of 66.7% and DCR of 100.0% were obtained in NSCLC patients with *ROS1* fusions without previous crizotinib treatment, and these results were similar to that obtained with crizotinib (ORR 72%) [17]. One patient receiving 800 mg had a CR and stayed on the treatment for more than 16 months, and some patients had reduced brain metastases.

In the four patients previously treated with crizotinib, one 34-year-old male patient, who achieved a PR, received DS-6051b as a third-line treatment. The two patients with SD, who discontinued treatment due to disease progression, previously received eight and 11 regimens, respectively. Therefore, even for crizotinib-pretreated *ROS1* fusion-positive patients, DS-6051b may be more effective as an earlier treatment line. However, due to our small sample size, we could not confirm that DS-6051b may offer a therapeutic option to help overcome crizotinib resistance.

A key limitation of this study was the small sample size. Of the 15 subjects enrolled, only four NSCLC patients with *ROS1* fusions had a history of crizotinib treatment. In addition, no patients had *NTRK* fusions; therefore, further clinical research is needed incorporating this population.

In conclusion, DS-6051b is well tolerated in Japanese patients with NSCLC and effective in crizotinib treatment-naïve patients. DS-6051b has antitumor activity and may be a therapeutic option for NSCLC patients with *ROS1* fusions. This advances the development of targeted therapies for the molecularly distinct *ROS1* fusion-positive subset of NSCLC patients.

## MATERIALS AND METHODS

### Patients

Adult (≥20 years) Japanese patients with histologically or cytologically confirmed solid tumors harboring either *ROS1* or *NTRK* fusions, refractory to standard therapy (or with tumors for which no standard therapy was available), were included in this study. Gene fusions were detected by fluorescence *in situ* hybridization, reverse transcription-polymerase chain reaction, next-generation sequencing, or other appropriate assays in local laboratories.

Patients were required to have attained treatment-free periods of at least 1 week after crizotinib therapy; at least 3 weeks for prior chemotherapy other than crizotinib, immunotherapy, or radiation therapy; and 4 weeks following cancer surgery. Patients with another malignant tumor requiring treatment, symptomatic or treatment-requiring brain metastasis, or any serious concomitant disease condition were excluded.

The study was conducted in compliance with the International Ethical Guidelines for Biomedical Research Involving Human Subjects, Good Clinical Practice Guidelines, the Declaration of Helsinki, and local laws. All patients provided written informed consent. The study protocol and any subsequent amendments were approved by the relevant institutional review boards or independent ethics committees.

### Study design, treatments, and blinding

This was a phase I, multicenter, non-randomized, open-label, multiple-dose study. Eligible patients received level 1 (400 mg QD, *n* = 6), level 2 (800 mg QD, *n* = 3), or an additional level (600 mg QD, *n* = 6) of oral DS-6051b treatment in cycles of 3 weeks until Response Evaluation Criteria in Solid Tumors (RECIST version 1.1)-defined disease progression or unacceptable toxicity was observed, in accordance with a pre-specified table of criteria for dose escalation, based on the presence/absence of DLTs. Concomitant use of another cancer therapy was not permitted during the study period.

### Objectives

The primary objective was to evaluate the safety and tolerability of DS-6051b in Japanese patients with solid tumors harboring either a *ROS1* or *NTRK* fusion. The secondary objectives were to determine the MTD (defined as the maximum dose at which the incidence of DLTs is <33%) and recommended phase II dose and PK of DS-6051b. Additionally, an exploratory evaluation was performed for biomarkers and tumor responses to DS-6051b.

### Safety

Safety was evaluated in all patients who received at least one dose of DS-6051b. DLTs were evaluated during cycle 1 (days 1–21). Additional assessments included laboratory tests, body weight, vital signs, Eastern Cooperative Oncology Group Performance Status, ophthalmological examination, and 12-lead electrocardiogram. All AEs were classified and graded by the Common Terminology Criteria for Adverse Events (CTCAE), version 4.0. Patients with AEs were followed up until resolution.

### PK analysis

Blood samples were collected and assayed at a central laboratory for plasma DS-6051a (the free form of DS-6051b) concentrations using pre-validated high-performance liquid chromatography–tandem mass spectrometry methods. Standard PK parameters (C_max_, T_max_, and AUC_0–24 h_) were determined on serial PK sample collection days (days 1 and 15 of cycle 1) using a non-compartmental analysis approach. AUC_0–24 h_ on day 15 was compared between this Japan study (J102) and the US phase I study (U101).

### Efficacy assessments

Efficacy assessments included best overall response, ORR, DCR, progression-free survival, time to response, duration of response, duration of SD, and maximum percent tumor reduction. Efficacy was evaluated by Response Evaluation Criteria in Solid Tumors version 1.1 criteria every 6 weeks until week 24, and every 12 weeks subsequently using computed tomography and magnetic resonance imaging scanning. Biomarker evaluation was conducted in two patients (Supplementary Material).

### Statistical methods

AEs were tabulated by event, relationship, and CTCAE grade. Summary statistics were calculated for safety parameters, PK profiles, and efficacy parameters.

## SUPPLEMENTARY MATERIALS TABLES



## Abbreviations

NSCLC: non-small cell lung cancer
ROS1: receptor tyrosine kinase gene
NTRK: neurotrophic receptor tyrosine kinase
ORR: objective response rate
PK: pharmacokinetics
RECIST: Response Evaluation Criteria in Solid Tumors
QD: once daily
DLTs: dose-limiting toxicities
MTD: maximum-tolerated dose
AEs: adverse events
CTCAE: Common Terminology Criteria for Adverse Events
SD: stable disease
DCR: disease control rate
AST: aspartate aminotransferase
ALT: alanine aminotransferase
PR: partial responses
CR: complete response
