# Supplementary material for: Safety and pharmacokinetics of DS-6051b in Japanese patients with non-small cell lung cancer harboring ROS1 fusions: a phase I study
Source: Oncotarget. 2018 May 4;9(34):23729–37. doi: 10.18632/oncotarget.25263 (PMC5955103; doi:10.18632/oncotarget.25263)
Supplement: Supplementary file 1 [file oncotarget-09-23729-s001.pdf]

Safety and pharmacokinetics of DS-6051b in Japanese patients with non-small cell lung cancer harboring *ROS1* fusions: a phase I study

SUPPLEMENTARY MATERIALS

Biomarker analysis

Biomarker evaluation was conducted in two patients who agreed to undergo this additional analysis. This included optional assessments for *ROS1* fusions in circulating tumor DNA (ctDNA) as liquid biopsies and individual genetic mutations in the biomarker analysis set.

The ctDNA detection rate was 100% (two samples tested). In one patient (#10), a *CD74-ROS1* fusion was detected, but no *de novo* secondary *ROS1* mutation was observed. No fusion was detected in the other patient (#15).

Of the two patients in the biomarker analysis, one was *ROS1* fusion-positive, with no *de novo* secondary *ROS1* mutation (#10), and was crizotinib-naïve. In the other, although a *ROS1* fusion was detected before registration, it subsequently was not detected in ctDNA and the possibility of a secondary *ROS1* mutation was unconfirmed. As there was no confirmation of a secondary *ROS1* mutation, which is thought to be related to crizotinib intolerance, and because crizotinib was discontinued due to AEs, this patient is likely to be crizotinib intolerant rather than crizotinib resistant.

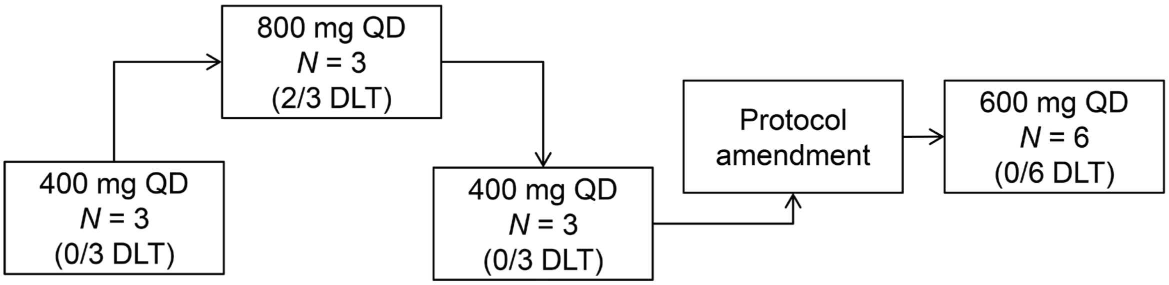

Supplementary Figure 1: Patient flow. DLT, dose-limiting toxicity; QD, once daily.

Supplementary Table 1: Pharmacokinetic profile of DS-6051b

		Day 1			Day 15			
**Dose (mg)**		**C_{max} (ng/mL)**	**T_{max} (h)**	**$AUC_{0–24\ h}$ (ng·h/mL)**	**C_{max} (ng/mL)**	**T_{max} (h)**	**$AUC_{0–24\ h}$ (ng·h/mL)**	**Vz/F**
400	Geometric Mean	268	3.4	3160	469	3.5	8770	3.02
(*n* = 6)	CV% Geometric Mean	24.4	32.9	22	24.6	36.7	27.7	29.1
	Min	183	2.0	2130	323	2.0	5660	2.08
	Median	268	4.0	3380	481	4.0	9060	2.99
	Max	356	5.0	3870	647	5.0	12200	4.81
	Range	170	3.0	1700	320	3.0	6500	2.7
600	Geometric Mean	205	3.2	2570	670	5.7*	13000	6.85
(*n* = 6)	CV% Geometric Mean	69.5	55.4	67.2	16.9	35.8*	22.6	406.5
	Min	67.7	2.0	1020	529	0.0	10100	2.56
	Median	241	3.0	3280	701	5.0	13200	3.15
	Max	374	8.0	4310	802	8.0	16300	86.3
	Range	310	6.0	3300	270	8.0	6200	84
800	Geometric Mean	427	5.4	5950	886	3.9	17500	3.78
(*n* = 3)	CV% Geometric Mean	22.2	36.5	5.9	10.3	37.3	9.4	33.7
	Min	332	4.0	5620	824	3.0	16300	2.99
	Median	473	5.0	5930	889	4.0	17500	3.88
	Max	496	8.0	6330	953	5.0	18700	4.76
	Range	160	4.0	700	130	2.0	2300	1.8

*n = 5.
